# Antagomir-mediated suppression of microRNA-134 reduces kainic acid-induced seizures in immature mice

**DOI:** 10.1038/s41598-020-79350-7

**Published:** 2021-01-11

**Authors:** Aoife Campbell, Gareth Morris, Janosch P. Heller, Elena Langa, Elizabeth Brindley, Jesper Worm, Mads Aaboe Jensen, Meghan T. Miller, David C. Henshall, Cristina R. Reschke

**Affiliations:** 1 Department of Physiology and Medical Physics, Royal College of Surgeons in Ireland, 123 St. Stephen’s Green, Dublin, D02 YN77 Ireland; 2 FutureNeuro, The SFI Research Centre for Chronic and Rare Neurological Diseases, Royal College of Surgeons in Ireland, Dublin, Ireland; 3 Queen Square Institute of Neurology, University College London, London, UK; 4 Roche Innovation Center Copenhagen, Copenhagen, Denmark; 5 F. Hoffmann-La Roche Ltd, Basel, Switzerland; 6 INFANT Research Centre, University College Cork, Cork, Ireland

**Keywords:** Neuroscience, Diseases of the nervous system, Epilepsy

## Abstract

MicroRNAs are short non-coding RNAs that negatively regulate protein levels and perform important roles in establishing and maintaining neuronal network function. Previous studies in adult rodents have detected upregulation of microRNA-134 after prolonged seizures (status epilepticus) and demonstrated that silencing microRNA-134 using antisense oligonucleotides, termed antagomirs, has potent and long-lasting seizure-suppressive effects. Here we investigated whether targeting microRNA-134 can reduce or delay acute seizures in the immature brain. Status epilepticus was induced in 21 day-old (P21) male mice by systemic injection of 5 mg/kg kainic acid. This triggered prolonged electrographic seizures and select bilateral neuronal death within the CA3 subfield of the hippocampus. Expression of microRNA-134 and functional loading to Argonaute-2 was not significantly changed in the hippocampus after seizures in the model. Nevertheless, when levels of microRNA-134 were reduced by prior intracerebroventricular injection of an antagomir, kainic acid-induced seizures were delayed and less severe and mice displayed reduced neuronal death in the hippocampus. These studies demonstrate targeting microRNA-134 may have therapeutic applications for the treatment of seizures in children.

## Introduction

Epilepsy is a common neurological disease caused by hyperexcitable and hypersynchronous brain networks that produce seizures. Seizures in infants and children are one of the most common neurological emergencies, and around 5% of children will have a seizure^1^. Status epilepticus (SE) is a prolonged seizure that has an incidence of 17–23 per 100,000 children^2,3^ and a 16% mortality rate^4^. Seizures at an early age may trigger significant developmental impairment and can result in an increased risk of developing epilepsy later in life^5,6^. Frontline seizure control is with anti-epileptic drugs (AEDs) and acute administration of anticonvulsants. Despite the availability of more than 20 different AEDs, over 20% of children remain refractory^7,8^. Furthermore, benzodiazepines are ineffective in controlling SE in almost 50% of cases^9,10^. Thus, there is a major unmet clinical need for new therapeutic approaches to treat paediatric seizures and protect the immature brain.

The functional properties of neuronal networks are a product of the rich diversity of genes expressed in the brain. A number of post-transcriptional mechanisms ensure tight regulation of protein levels, including local control of protein synthesis at synapses^11,12^. MicroRNAs (miRNAs) are small (~ 21 nt) non-coding RNAs that play a crucial role in the post-transcriptional negative regulation of gene expression. After uptake by Argonaute proteins (Ago), miRNAs perform sequence-specific targeting of protein-coding mRNAs resulting in transcript degradation or translation inhibition. Various miRNAs have been identified to have a role in normal brain development and function^13^. Dysregulation of miRNAs has been implicated in the pathogenesis of a number of neurological diseases, including epilepsy^14^. Accordingly, miRNAs have emerged as important new treatment targets^14–16^. Among these, miR-134, a brain-enriched miRNA, has been found to be upregulated in the hippocampus of children^17^ and adults^18^ with mesial temporal lobe epilepsy (MTLE), and increased in the same brain structures following experimentally-induced seizures in rodent models^17–21^. Several targets of miR-134 have been identified, including Lim kinase 1 (Limk1), a protein involved in dendritic spine dynamics^22^ and doublecortin (DCX)^23^. Functional studies showed that decreasing brain levels of miR-134 with a locked nucleic acid (LNA) oligonucleotide antagomir (Ant-134) reduced severity or delayed seizure onset in multiple adult rodent models and protected against attendant brain damage^17,18,21^. Ant-134 may also have disease-modifying effects when given after SE, reducing the later occurrence of spontaneous recurrent seizures in both mice and rats^18,21,24^.

The potent in vivo anti-seizure effects of Ant-134 have only been demonstrated in adult models of epilepsy^18,19,21,24^. Given that miR-134 is also dysregulated in children with epilepsy^17^, we hypothesized that targeting miR-134 using Ant-134 could protect against seizures in the immature brain. Such evidence would broaden the potential range of therapeutic applications of this approach to seizure control. However, the transcriptomic landscape and patterns of gene expression change during brain maturation and this could reshape the target pool of a miRNA, leading to alternate cellular functions. Here we profiled miR-134 expression during postnatal development, investigated the effects of seizures on the expression of miR-134 in the immature mouse hippocampus and tested the effects of Ant-134 in a paediatric seizure model. We show that Ant-134 can reduce seizures in juvenile mice in a highly dose-specific manner and may be related to de-repression of DCX.

## Results

### miR-134 expression in mouse brain during postnatal development

**Figure 1:**
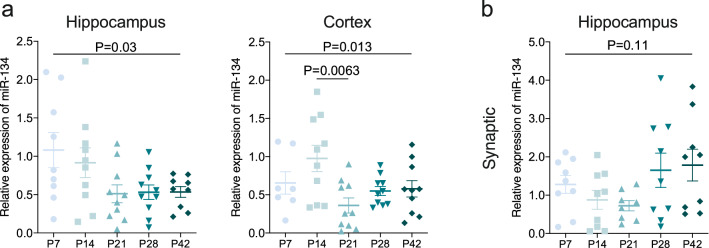
Expression of miR-134 in naïve brain over the developmental period P7-P42. (**a**) Overall miR-134 levels in naïve hippocampus and neocortex following standard RNA extraction (N = 10/group), overall change of levels of miR-134 over developmental period following standard RNA extraction with a significant decrease in cortex between P14 and P21. (**b**) Synaptic miR-134 levels in hippocampus. Two hippocampi were pooled to make up each experimental number (N = 9 per group for hippocampus), no change in levels of miR-134 following synaptic enrichment.

We first sought to establish the expression of miR-134 during normal postnatal brain development in mice (P7, P14, P21, P28) compared to the levels in the adult (P42) brain. Analysis of mature miR-134 expression over the postnatal period, using total RNA extracted from tissue homogenates, found quite variable expression at the earliest time points and a slight decrease in hippocampal miR-134 levels with age (Fig. 1a; N = 9 [P7, P42] or 10 [P14, P21, P28] per group; one-way ANOVA—overall *P* = 0.030). Cortical miR-134 expression also varied over development (Fig. 1a; N = 7 [P7] or 10 [P14, P21, P28, P42] per group; one-way ANOVA—overall *P* = 0.013), with a small decrease detected between P14 and P21 (Bonferroni *posthoc* test; *P* = 0.0063).

Due to the known enrichment of miR-134 in dendrites^22^ we also explored whether any developmental differences were evident in this compartment by analysing synaptically-enriched fractions from pooled hippocampi of mice. As expected, miR-134 was readily detected in the synaptic fractions of immature mouse brain. However, synaptic miR-134 expression did not change significantly throughout development (Fig. 1b; N = 9 [P21] or 10 [P7, P14, P28, P42] per group; one-way ANOVA—overall *P* = 0.11). These findings suggest that miR-134 expression in the mouse brain can vary during the post-natal period in certain subcellular compartments and brain structures.

### Characterisation of kainic acid-induced seizures in P21 mice

**Figure 2:**
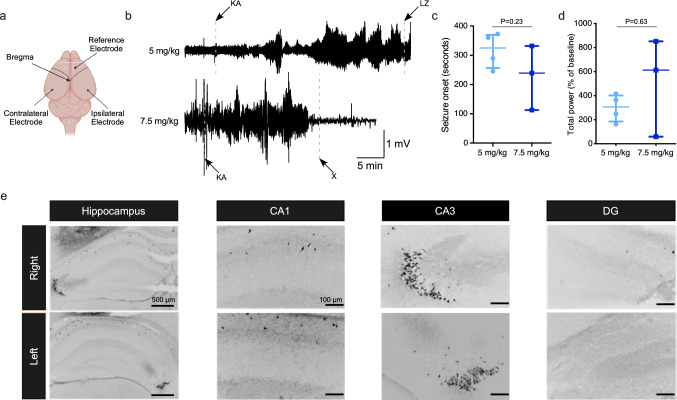
KA dose selection for the induction of seizures in P21 mice. (**a**) Schematic of electrode placement in P21 mouse brain. (**b**) Representative EEG traces for mice injected with 5 or 7.5 mg/kg KA (KA—KA injection, LZ—lorazepam injection, X—mouse died). (**c**) Graph shows no difference in onset to first electrographic seizure after the IP injection of 5 (N = 4) or 7.5 (N = 3) mg/kg of KA. (**d**) Graph shows no difference in % of increase in EEG total power, relative to baseline, recorded over the 30 min after KA injection. (**e**) Representative images of FJB staining show neuronal death at 24 h post SE (5 mg/kg KA) in the right and left hippocampi of a P21 mouse.

To model seizures in the developing brain, we administered a systemic (intraperitoneal; IP) injection of kainic acid (KA) in P21 mice, based on previous studies^25,26^ and recorded the resulting electrographic activity using intracranial electroencephalography (EEG) (Fig. 2a,b). P21 mice are thought to approximate early adolescence in humans^27^. During preliminary dose range-finding studies, 7.5 mg/kg KA caused robust and reproducible seizures at this age (Fig. 2b). The median seizure onset after 7.5 mg/kg KA was 239 s (Interquartile range [IQR]: 112.9–331.6 s; Fig. 2c) and total EEG power was increased to 613% (median; IQR: 59.3–851.9%) from the baseline recordings (Fig. 2d). However, this dose of KA was associated with high mortality (3 of 3 mice within 24 h) and was therefore discontinued. A lower dose of KA (5 mg/kg) also resulted in reliable induction of SE (Fig. 2b–d) but with lower mortality (in 1 of 4 mice tested). Mice that received 5 mg/kg KA developed the first electrographic seizure after 325 s (IQR: 256.3–369.6; Fig. 2c) and had total EEG power of 306% (IQR: 185.3–402.2%) of baseline (Fig. 2d). We did not see any significant differences between electrographic activity elicited by 5 mg/kg or 7.5 mg/kg KA (Seizure onset: Mann–Whitney U test, *P* = 0.23, Fig. 2c; Total power: Mann–Whitney U test, *P* = 0.63, Fig. 2d).

We next assessed whether the seizures in this model induced brain damage. Fluoro-Jade B (FJB) staining of brain tissue sections obtained 24 h after SE induced by 5 mg/kg KA revealed typical patterns of KA-induced neuronal death in the hippocampus, primarily in the CA3 subfield (Fig. 2e). While always present, the extent and distribution of damage varied between hippocampal hemispheres, as is common with systemic administration of KA^28^. Taken together, these results support the induction of SE in P21 mice by 5 mg/kg IP KA as a reliable model to study the effects of miR-134 manipulation on seizures in the immature brain.

### miRNA levels following KA-induced seizures in P21 mice

**Figure 3:**
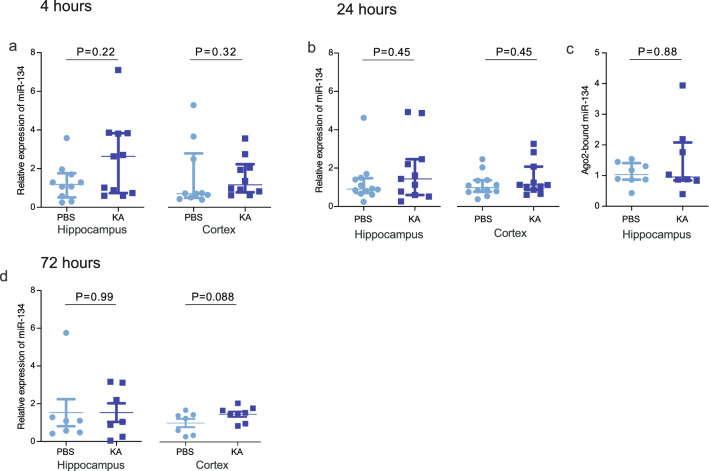
Systemic KA does not alter miR-134 expression in P21 mice. Graphs show real-time quantitative PCR analysis of miR-134 expression in hippocampal and cortical lysates at different time points after systemic KA injection, with counts normalised to RNU6B. (**a**) Overall miR-134 expression at 4 h (N = 10–11/group). (**b**) Overall miR-134 expression at 24 h (N = 11–12/group). (**c**) Ago2-bound (functionally engaged) miR-134 in hippocampus at 24 h (N = 8/group). (**d**) miR-134 expression at 72 h (N = 7/group).

We next explored whether acute seizures induced by systemic KA altered the expression of miR-134 in P21 mice. RT-qPCR analysis of hippocampal and cortical samples obtained 4, 24 or 72 h after seizures revealed no significant changes in mature miR-134 levels in either structure at any time point (Fig. 3a,b,d; Table 1). To confirm that subtle changes in the functional miRNA pool were not missed in the total miRNA analyses, we measured levels of miR-134 at 24 h after SE in a complex with Ago2, which guides the miRNA silencing complex to target mRNAs^29^. Ago2 was immunoprecipitated from mouse hippocampus after seizures and bound miRNA eluted and quantified. Again, no changes were observed in the level of Ago2-bound miR-134 in samples 24 h after KA-induced seizures (Fig. 3c; PBS: 1.04(0.87–1.41) N = 8; KA: 0.96(0.84–2.08) N = 8; Mann Whitney U test, *P* = 0.88). Together, these findings suggest that systemic KA-induced seizures in P21 mice do not change overall levels or the functional pool of miR-134 within affected brain structures.

**Table 1:** Statistical details for miR-134 expression after status epilepticus (Fig. 3).

Timepoint	Region	PBS (AU)	KA (AU)	Statistical test	*P* Value
4 h	Hippocampus	1.19 (0.51–1.77) (n = 10)	2.63 (0.74 3.84) (n = 11)	Mann Whitney U	0.22
4 h	Cortex	0.71 (0.49–2.79) (n = 10)	1.16 (0.78–2.23) (n = 10)	Mann Whitney U	0.32
24 h	Hippocampus	0.90 (0.70–1.47) (n = 12)	1.44 (0.60–2.50) (n = 11)	Mann Whitney U	0.45
24 h	Cortex	0.99 (0.76–1.37) (n = 12)	1.12 (0.87–2.08) (n = 11)	Mann Whitney U	0.45
72 h	Hippocampus	1.09 (0.48–1.29) (n = 7)	1.04 (0.25–3.12) (n = 7)	Mann Whitney U	0.99
72 h	Cortex	1.21 ± 0.26 (n = 7)	1.77 ± 0.17 (n = 8)	Unpaired *t*-test	0.088

Average values reported as Median (IQR) for non-parametric and mean ± SEM for parametric comparisons.

### Ant-134 suppresses levels of miR-134 in the hippocampus

Since upregulation of miR-134 may not be necessary for Ant-134 to have anti-seizure effects^21^, we proceeded to test whether reducing levels of miR-134 could protect against acute KA-induced seizures in P21 mice. We first sought to establish suitable dosing of Ant-134 to reduce miR-134 levels in naïve P21 mice. Mice received a single intracerebroventricular (ICV) injection of 0.05, 0.1 or 0.5 nmol Ant-134 and levels of miR-134 were assessed 24 h later.

**Figure 4:**
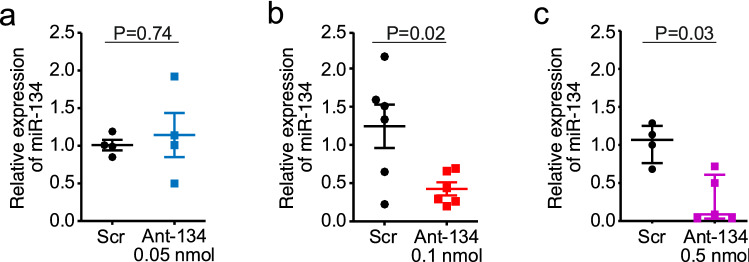
Ant-134 silences miR-134 in the hippocampus of naïve mice. (**a**) Relative expression of miR-134 following ICV injection of Scr/Ant-134 (0.05 nmol), (N = 4/group). (**b**) Relative expression of miR-134 following ICV injection of Scr/Ant-134 (0.1 nmol), (N = 6 [Scr] or N = 6 [Ant-134]. (**c**) Relative expression of miR-134 following ICV injection of Scr/Ant-134 (0.5 nmol), (N = 4 [Scr] or N = 5 [Ant-134]).

Ant-134 caused a dose-dependent knockdown of miR-134 in the hippocampus of P21 mice (Fig. 4a). At the lowest dose, 0.05 nmol Ant-134 produced only variable and non-significant knockdown of miR-134 (N = 4, Mann–Whitney U, *P* = 0.74). In contrast, 0.1 nmol Ant-134 significantly reduced miR-134 levels to ~ 65% of control (N = 6*, t*-test *P* = 0.020, Fig. 4b). 0.5 nmol Ant-134 produced a near-complete reduction in miR-134 levels in the hippocampus of P21 mice (N = 4 [Scr] or N = 5[Ant-134], Mann–Whitney U, *P* = 0.032, Fig. 4c).

### Ant-134 has dose-dependent protective effects against kainic acid-induced seizures in P21 mice

**Figure 5:**
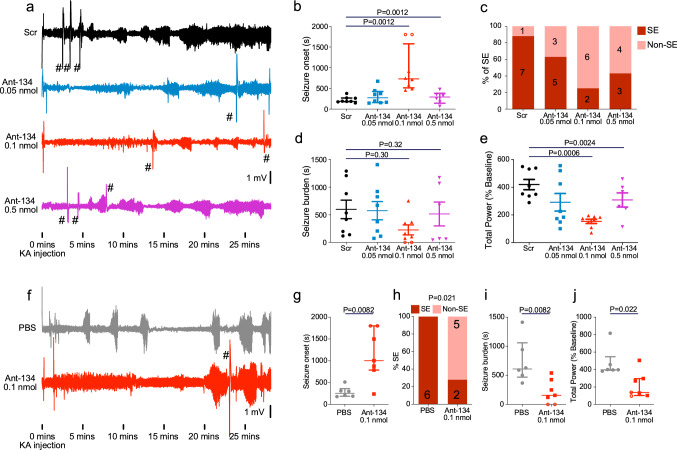
Protective effects of Ant-134 on SE severity in P21 mice. (**a**) Representative EEG traces from mice pre-treated with 0.05, 0.1 or 0.5 nmol Ant-134 in 2 µL PBS, or scramble control (Scr). Traces shown begin at the time of KA injection. (**b**) Dose-dependent effects of Ant-134 on seizure severity (N = 8 per group [Scr,0.05 nmol,0.1 nmol] or N = 5 [0.5 nmol]). 0.1 nmol Ant-134 increased time to seizure onset after KA injection. (**c**) There is a dose-dependent effect of Ant-134 on the percentage of mice experiencing SE following KA injection. There was no difference in seizure burden between groups and (**d**), however, there was a significant reduction in total EEG power in mice which received 0.1 nmol Ant-134 (**e**). (**f**) We performed a second set of experiments to validate the use of 0.1 nmol Ant-134 (N = 7, PBS N = 6). Representative traces start at the time of KA injection and show EEG recorded from mice pre-treated with 0.1 nmol Ant-134, or PBS control. (**g**) 0.1 nmol Ant-134 delayed seizure onset, reduced the percentage of mice experiencing full SE (**h**), reduced seizure burden, and reduced total EEG power (**j**). #—Artefacts caused by injections in other mice recorded simultaneously. Open circles represent mice which did not experience any seizure activity. Seizure onset was assigned a maximum value of 1800s for statistical purposes.

We next assessed the effects of lowering miR-134 levels on seizures induced by systemic KA in P21 mice. Mice received ICV injections of Ant-134 to silence miR-134 and were then subjected to KA-induced seizures 24 h later (Fig. 5a). Mice injected with 0.05 nmol Ant-134 experienced similar seizures to control animals injected with a scrambled oligonucleotide, in line with negligible miR-134 knockdown seen with this dose (Fig. 5a). In contrast, mice pre-treated with 0.1 nmol Ant-134 displayed a significant delay to the first electrographic seizure (Fig. 5b, N = 5 [0.5 nmol] or N = 8 [Scr, 0.05 nmol, 0.1 nmol] per group; Kruskal–Wallis with Dunn’s *posthoc* testing; Overall *P* = 0.0012; Scr vs 0.1 nmol *P* = 0.0012), with six out of eight mice not developing full SE during the 30 min recording period (Fig. 5c). There was no difference in the seizure burden time in mice treated with Scr and different doses of Ant-134 (Fig. 5d). Mice treated with 0.1 nmol Ant-134 also displayed reduced electrographic seizure severity (Fig. 5e, N = 5 [0.5 nmol] or N = 8 [Scr, 0.05 nmol, 0.1 nmol] per group; One-way ANOVA with Bonferroni posthoc test; Overall *P* = 0.0024; Scr vs 0.1 nmol *P* = 0.0006). At the highest tested dose, 0.5 nmol Ant-134, mice were not protected against KA-induced seizures (Fig. 5a,b) and high mortality (100%) was observed. We pursued potential explanations for why the higher dose did not show protection, staining tissue sections from 0.5 nmol-injected mice for markers of gliosis. This revealed minimal differences between Ant-134 and Scr treated mice, although there was a suggestion of some astrogliosis, indicated by GFAP staining, in the cortex (Mann–Whitney U, *P* = 0.048). Iba-1 staining, a marker of microgliosis, was not changed by 0.5 nmol Ant-134, and both GFAP and Iba1 were unaffected in the hippocampus and other regions including the amygdala examined (Supplementary Fig. S1, N = 3 mice per group). Additionally, mice injected with 0.5 nmol Ant-134 did not exhibit any visual signs of toxicity (e.g. differences in body weight, changes in neurological reflexes for the developmental stage or locomotor behaviour in their home cages). These results suggest a narrow tolerance for miR-134 inhibition only when associated with KA induction of seizures in P21 mice.

We next sought to validate the seizure suppressive effects of Ant-134 at the 0.1 nmol dose in a second independent cohort of mice, using a different batch of Ant-134 (Fig. 5f–j). As before, ICV injection of 0.1 nmol Ant-134 resulted in delayed seizure onset when P21 mice were exposed to systemic KA the next day (Fig. 5g; N = 6 [PBS] or N = 7 [0.1 nmol]; Mann–Whitney U test; *P* = 0.0082). Five out of seven mice did not develop full SE during the recording period, compared with zero out of six in control mice (Fig. 5h, Fisher’s exact test, *P* = 0.021). Further, we observed significant reductions in seizure burden (Fig. 5i; N = 6 [PBS] or N = 7 [0.1 nmol]; Mann–Whitney U test; *P* = 0.0082) and total EEG power in 0.1 nmol-injected P21 mice after KA injection (Fig. 5j; N = 6 [PBS] or N = 7 [0.1 nmol]; Mann–Whitney U test; *P* = 0.022), confirming that the pre-treatment with 0.1 nmol Ant-134 has seizure-suppressive effects in this model. We observed no difference in electrographic activity between control and Ant-134 treated mice following administration of lorazepam (Supplementary Fig. S2; N = 6 [PBS] or N = 7 [0.1 nmol]; Mann–Whitney U test; *P* = 0.84).

### Ant-134 has protective effects in the hippocampus and de-represses cortical DCX

**Figure 6:**
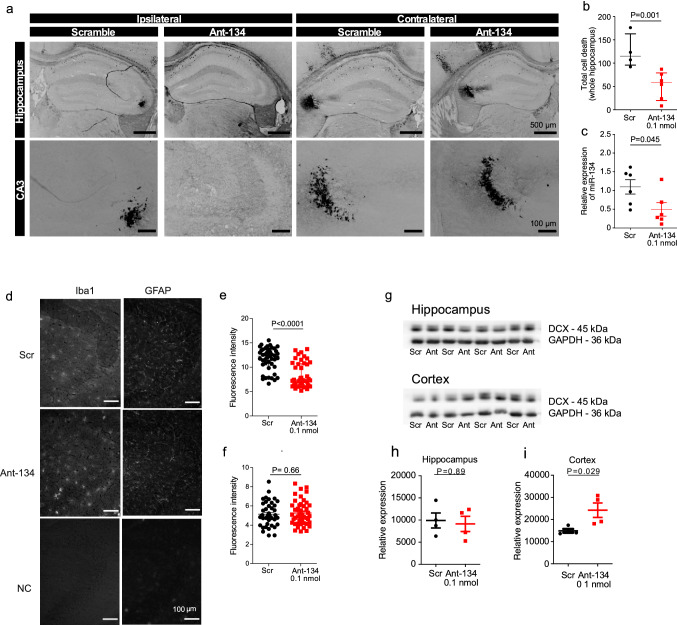
Ant-134 has protective effects in the hippocampus and de-represses cortical DCX. (**a**) Representative FJB-stained images show neuronal death in the hippocampus 24 h after SE ipsi- and contralateral to pre-treatment with 0.1 nmol Ant-134 or scramble control (Scr). (**b**) The number of FJB-stained cells was reduced in mice pre-treated with Ant-134 (N = 6) compared with scramble control (N = 4). (**c**) Partial knockdown of miR-134 levels (54.8%) was retained 24 h after SE in the hippocampus of mice that were pre-treated with 0.1 nmol Ant-134 (N = 6) or Scr (N = 6). (**d**) Representative immunohistochemistry staining with Iba1 and GFAP in the CA3 of the hippocampus after SE. (**e**) The number of activated microglia was decreased in mice pre-treated with Ant-134 (N = 3) compared with scramble control (N = 3). (**f**) There was no difference in the number of astrocytes in mice pre-treated with Ant-134 (N = 3) and scrambled control (N = 3). (**g**) Western blot showing DCX expression in hippocampus and cortex at 24 h after KA-induced seizures of mice pre-treated with Scr/Ant-134 (0.1 nmol). Full-length blot can be found in Supplementary Fig. S3a. (**h**) Densitometry analysis of DCX in the hippocampus. GAPDH was used as the loading control, (N = 4/group). (**i**) Densitometry of DCX in the cortex. GAPDH was used as the loading control (N = 4/group).

Finally, we assessed the effect of 0.1 nmol Ant-134 on neuronal death following SE in P21 mice. This was done using mice from the dose response study. Neuronal death was reduced in the hippocampus in 0.1 nmol Ant-134-treated P21 mice relative to Scr-injected controls (Fig. 6a,b, N = 4 [Scr] or N = 6 [0.1 nmol]; Mann–Whitney U test; *P* = 0.001). The neuroprotective effect was most pronounced in the hippocampus ipsilateral to Ant-134 injection (Fig. 6a). Patterns of protection in the hippocampus were variable, however; in some mice the CA3 subfield was entirely protected by 0.1 nmol Ant-134 pre-treatment. We also confirmed that miR-134 knockdown by 0.1 nmol Ant-134 persisted after SE (Fig. 6c; N = 6 [Scr and 0.1 nmol]; unpaired *t*-test; *P* = 0.045). We next investigated if 0.1 nmol Ant-134 protected against gliosis, focusing on the CA3 subfield of the hippocampus as the main site of damage. Mice that received 0.1 nmol Ant-134 had a lower numbers of activated microglia, as analysed by Iba1 staining, compared to Scr-treated seizure mice (Fig. 6d,e, N = 3 [Scr] or N = 3 [0.1 nmol]; Mann–Whitney U test; *P* < 0.0001). Levels of astrogliosis, as analysed by GFAP staining was similar between treatment (Fig. 6d,f, N = 3 [Scr] or N = 3 [0.1 nmol]; t-test; *P* = 0.659).

Finally, we sought insight into the mechanism of Ant-134. In a separate cohort of mice subjected to KA-induced seizures, cortical levels of DCX were higher in 0.1 nmol Ant-134 mice compared to Scr consistent with de-repression of this validated target of miR-134 (Fig. 6g,i, t*-*test, N = 4, *P* = 0.029). However, these changes were not present in the hippocampus (Fig. 6g,h, t-test, N = 4, *P* = 0.89). Levels of two other miR-134 targets appeared unchanged (Supplementary Fig. S3). Taken together, these data show that knockdown of miR-134 by Ant-134 can protect against chemoconvulsant-induced SE and subsequent neuronal death in the developing brain.

## Discussion

miRNA have emerged as promising new targets for the treatment of epilepsy and silencing miR-134 has been repeatedly demonstrated to have potent anti-seizure effects in the adult rodent brain^18,21^. The present study shows that inhibition of miR-134 using antisense oligonucleotides, within a narrow dose range, can protect against KA seizures in immature, P21 mice. Ant-134 delayed the onset of the first seizure, reduced the overall seizure severity and attenuated neuronal death after systemic KA administration. In addition, we report the hippocampal and cortical expression of miR-134 in mice over the developmental period and investigate the effects of seizures on miR-134 levels in the model. Together, these results are proof-of-concept that lowering miR-134 levels can protect against seizures in the immature brain.

There remains a major unmet need for novel approaches to treat seizures in the immature brain. miRNAs have emerged as important regulators of the gene expression landscape underlying brain structure and function, and their targeting, particularly using antisense oligonucleotides, has emerged as a promising therapeutic strategy for seizure control and disease modification^14^. The main focus to date, however, has been targeting miRNAs in the adult brain^14,18,21,30^. In particular, inhibition of miR-134 has been shown to suppress evoked and spontaneous seizures in adult rodents^18,21^. Expression of miR-134 has also been reported to be increased in brain tissue following SE in immature rodents and in resected brain samples from children with MTLE^17^. The present study is the first to functionally test whether inhibiting miR-134 in vivo can reduce seizures in the immature brain.

Here we show that inhibiting miR-134 using an antisense oligonucleotide, termed Ant-134, reduced acute seizures induced by systemic KA injection in P21 mice. While there remain challenges with how to age-match rodent to human brain development, the P21 mouse is likely to correspond to approximately early adolescence^31^. This is an important period of brain maturation, during which significant synaptic pruning and synaptogenesis occurs in both humans^31^ and mice^32,33^. The model we used was adopted from previous work in mice^25^ and rats^26^, and we observed similar electrographic seizure activity, comprising mainly bi-lateral electrographic seizures that started within a few min of KA injection and continued thereafter. We also observed bi-lateral damage to the hippocampus in the model. Together, this provides an age-appropriate model for an initial screen for potential anti-seizure effects of Ant-134 in the immature brain.

The most important finding of the present study was that pre-treating P21 mice with Ant-134 resulted in significant protection against KA-induced seizures. At a dose of 0.1 nmol, Ant-134 -treated mice displayed a delay to first seizure and reduced total EEG power, a measure of seizure severity. This matches the effects of Ant-134 at a similar dose in studies in which seizures were reduced in adult mice treated with intraamygdala KA^18^ and pilocarpine^19^ Thus, the effective anti-seizure dose for Ant-134 is similar regardless of age and model. The anti-seizure effects of Ant-134 appear to be superior when compared with another miRNA targeting strategy in juvenile rodents, where over-expression of miR-146a in P21-P28 rats only increased the latency to SE by roughly 20% in a lithium-pilocarpine model^34^.

The anti-seizure effects of Ant-134 were apparent despite the observation that miR-134 was not upregulated following SE in P21 mice. While an increase in miR-134 levels is commonly reported after seizures^17,18^, we have previously reported that this is not required for the anti-seizure effects of Ant-134. For instance, Ant-134 potently reduces spontaneous recurrent seizures in rats despite unchanged miR-134 levels in the perforant pathway stimulation model^21^. Currently, the role of miR-134 in the immature brain is incompletely understood. It is unclear why KA seizures did not change transcription of miR-134 levels in the P21 mice but may be due to generalised or lower intensity seizures compared to those generated by either intraamygdala KA^18^ or systemic pilocarpine^17,19^. Sampling timing may be a factor or levels may change within specific subfields or cell types. Since miRNA processing activity is stimulated by synaptic activity, it is possible that maturation differences in pathways such as those mediated by NMDA receptors^35^ could explain the failure of seizures to elicit changes in mature miR-134 levels. Together, these findings demonstrate that upregulation of miR-134 is not a pre-requisite for the anti-seizure effects of Ant-134 in immature mice, broadening the range of anti-seizure therapeutic applications. These acute effects of Ant-134 suggest that future studies into the long-term effects of Ant-134 during epileptogenesis in juvenile rodents would be beneficial.

We observed significantly less cell death in mice pre-treated with 0.1 nmol Ant-134 before KA administration, presumably due to seizure suppression. This protection was mainly within the vulnerable CA3 subfield of the hippocampus and was most evident ipsilateral to the side of Ant-134 injection. Because the anti-seizure effects were observed bi-laterally, this suggests a potential direct neuroprotective effect that might be local dose-dependent. This is consistent with evidence of direct neuroprotective effects of inhibiting miR-134 in hippocampal neurons in vitro^18^. Further studies will be needed to separate potential direct from indirect neuroprotective effects of Ant-134 in this model. In addition to a reduction in cell death, we also observed a reduced number of activated microglia in the CA3 of the hippocampus in Ant-134 treated mice. Since microglia perform important scavenging roles after brain injury, this is consistent with Ant-134 neuroprotection but could have longer-term consequences since microglia mediate potent epileptogenic actions^36^. The lack of difference in astrogliosis was unexpected and suggests astrocyte responses may be delayed relative to microgliosis^37^ or less sensitive to differences in neuronal death in the model.

A caveat of the approach we took for delivery of Ant-134 was to pre-treat mice 24 h before KA administration to determine how miR-134 inhibition affects seizure onset and development. This was necessary because antagomirs take at least 12 h to produce noticeable reductions in miRNA levels in the mouse hippocampus and then a further period of time must elapse for any targets to be de-repressed for a phenotype to emerge^18^ . While this approach allowed us to demonstrate that a lower level of miR-134 reduces seizures in the immature brain, it does not reflect a clinically-relevant strategy. Future studies, delivering Ant-134 after seizures have commenced, or once animals fail to respond to a frontline anticonvulsant, would allow us to explore whether targeting miR-134 has any delayed or late anti-seizure or disease-modifying effects.

In the present study we observed an apparently narrower range of safe and effective doses of Ant-134 for seizure control when compared to the adult rodent brain. Specifically, injection of a dose of Ant-134 that resulted in near-complete knockdown of miR-134 was associated with seizure-related mortality. Administration of the same dose of Ant-134 in naive mice did not appear to trigger toxicity, suggesting a context-dependent effect of miR-134 knockdown. Since dose-related toxicity was not reported in adult studies^18^, this may be due to loss of a function of miR-134 that plays a key role in seizure pathogenesis in the developing brain. Notably, several miR-134 targets are critical for brain development. These include the neuronal guidance molecule DCX^23,38^, which we observed to be de-repressed in the cortex following Ant-134 administration, and the RNA-binding protein Pumilio2 (Pum2)^39^, which is implicated in activity-dependent dendritogenesis. These dose-dependent effects of Ant-134 are consistent with a ‘tuning model’ in which miRNA levels are required to be modulated to within a certain range^38^ and excessive over- or under-expression can be deleterious. Further studies will be required to determine whether this is specific to this KA model, mouse age or genetic background.

The mechanism of action of Ant-134 is currently unknown. The pronounced anti-seizure effects are supportive of an effect on neuronal network synchrony and recent work reported Ant-134 does not alter basic electrophysiological properties of principal neurons in the hippocampus^20^. Limk1 is a validated target of miR-134 in the rodent brain and inhibition of miR-134 alters dendritic spine morphology whereas depleting Limk1 obviates the neuroprotective effects of Ant-134 in in vitro models of seizure-induced neuronal death^18^. However, miR-134, like other miRNAs, has an extensive repertoire of targets and this will likely change during brain maturation. This might be resolved by sequencing the transcriptional landscape of the brain after treatment with Ant-134 and accompanying functional studies to interrogate other age-relevant targets such as DCX^23^. Here, we show that miR-134 inhibition de-repressed levels of DCX in the cortex, and, notably, loss of DCX is associated with epilepsy in mice and humans^40^. Levels of Limk1 and CREB remained unchanged in the hippocampus and cortex of Ant-134-treated mice and we cannot exclude that the anti-seizure mechanism could involve different targets in immature and adult brain^18^.

In summary, the present study demonstrates that inhibition of miR-134 can reduce seizures and attendant brain damage in the immature mouse brain. Seizures in this model did not upregulate miR-134, and dose-ranging experiments may indicate a narrower therapeutic window for the use of this miRNA inhibitor than in adults. In summary, moderate knockdown of miR-134 can reduce seizures in P21 mice, expanding the potential applications of this novel approach to the treatment of seizures and epilepsy. Finally, we note that more than a dozen miRNAs have been reported as potential targets for seizure control in the adult brain^14^. It is likely, therefore, that miRNAs in addition to miR-134 will provide a rich set of anti-seizure targets for investigation.

## Materials and methods

### Animals

All animal experiments were performed in accordance with the European Communities Council Directive (86/609/EEC) the NIH Guide for the Care and Use of Laboratory Animals, and followed ARRIVE guidelines. Protocols were reviewed and approved by the Research Ethics Committee of the Royal College of Surgeons in Ireland (REC 1302bbb) under license from the Department of Health (AE19127/P013), Dublin, Ireland. Male C57BL/6OlaHsd mice (P7-42) were obtained from RCSI’s biomedical research facility (original stock from Harlan, Oxon, Bicester, U.K.). P7 and P14 mice were housed with littermates and dam until non-recovery experiments. Weaned animals were housed (up to 5 mice per cage) in on-site barrier-controlled facilities with a 12 h light–dark cycle and ad libitum access food and water.

### Electrode implantation and intracerebroventricular (ICV) injections

Mice were anesthetised with isoflurane (5% induction, 1–2% maintenance) and placed in a mouse-adapted stereotaxic frame. After local analgesia with lidocaine and prilocaine cream (EMLA), a midline scalp incision was made, Bregma was located and three partial craniotomies were performed (two cortical electrodes placed behind bregma and the reference electrode was placed towards the front of the brain, above bregma on the right hand side). Schematic drawing can be found in Fig. 2a for the placement of skull-mounted recording screws (Bilaney Consultants, Kent, UK) for intracranial electroencephalographic (EEG) recordings. Where necessary, a fourth craniotomy was drilled to allow direct ICV injections (coordinates from Bregma: anterior–posterior (AP) + 0.3 mm, lateral (L) =  + 0.9 mm, ventral (V) = − 1.35 mm. Mice received either mmu‐miR‐134‐5p miRCURY LNA power inhibitor (Ant-134; Exiqon; 0.05–0.5 nmol in 2 µL PBS), a non-targeting scrambled control (Scr; Exiqon, 0.5 nmol in 2 µL PBS), or vehicle control (2 µL PBS), injected using a 2 µL Hamilton syringe at a rate of 1 µL/min. The electrode assembly was fixed in place using dental cement. After surgery, the mouse was placed in a temperature-controlled open Perspex box and monitored throughout post-op recovery.

### EEG recording during KA-induced seizures

Two hours after the surgical procedure, mice were evaluated and considered fully recovered when moving freely and eating without mobility issues. Following full recovery, mice were connected to the lead socket of a swivel commutator, which was connected to a brain monitor (Xltec, 32 channels, Natus Neurology) amplifier for acute tethered EEG recordings. The short recovery timing was due to the need to perform all experiments on the same postnatal day (P21). Instrumentation of mice before P21 to allow a recovery week was not undertaken because of the challenges with operating on pre-weaned animals with active skull and brain growth. After a 15 min EEG baseline recording, mice were lightly restrained while KA (Sigma-Aldrich, 5 mg/kg or 7.5 mg/kg intraperitoneal; IP) was injected to induce seizures. After 30 min, mice received lorazepam (LZ; 8 mg/kg, IP) to terminate seizures and reduce morbidity and mortality, and EEG was recorded for a further 15 min. Mice were then disconnected from the EEG system and placed in a warmed recovery chamber. EEG data were analysed and quantified manually using LabChart 8 software (AD instruments, Oxford, UK) by an observer blind to the experimental groups. Seizures were defined as high-amplitude (> 2 × baseline) high-frequency (> 5 Hz) polyspike discharges lasting > 5 s. Status epilepticus was defined as at least 5 min of continuous seizure activity without a return to baseline. The presence of SE, the onset to first electrographic seizure, the total time spent in seizure/SE (seizure burden) and the total EEG power (0–40 Hz, measured by fast Fourier transform) between KA and LZ injections were recorded for each mouse. Mice that did not present electrographic seizures during the recording period were assigned cut-off time as onset (1800 s) for statistical purposes. Clinical and convulsive behavior was not scored in mice because use of tethered EEG in animals of this age restricted clear assessment. The surgical procedures, the induction of seizures and EEG analysis were performed by different researchers whom were blind to the treatment. With the exception of mice euthanized 4 and 72 h after KA injection, all mice were euthanized and brain samples were collected at 24 h after KA injection to follow with in vitro assessments.

### Quantification of miRNA levels

For the analysis of miR-134 expression following KA-induced seizures, mice received an intraperitoneal overdose of pentobarbital 24 h after KA and were transcardially perfused with ice-cold PBS to remove the intravascular blood components. Brains were removed and microdissected to isolate the hippocampus and cortex for molecular analyses.

### Synaptoneurosome preparation

Method was adapted from Nagy and Delgado-Escueta^41^. Two hippocampi were pooled per mouse. Hippocampi were homogenised on ice in 2 mL of cold homogenising buffer with added protease and phosphate inhibitors. Samples were centrifuged for 10 min at 3000 g and the supernatant was recovered. The supernatant was centrifuged at 14,000 *g* for 12 min. and the cytosol was removed. The pellet was re-suspended in 200 µL of Krebs–Ringer buffer, followed by 90 µL of Percol (45% v/v) and centrifuged at 22,000 *g* for 2 min. The enriched synaptoneurosomes were recovered and re-suspended in 1 mL of Krebs–Ringer buffer. The samples were centrifuged at 22,000 *g* for 30 s and the supernatant was discarded. Finally, the pelleted synaptoneurosomes were re-suspended in Trizol and a standard RNA extraction for low input was carried out.

### Argonaute-2 immunoprecipitation

Hippocampi were homogenised by hand in 500 µL immunoprecipitation buffer [150 mM NaCl, 20 mM Tris–HCL (pH 7.5), 5 mM MgCl_2_, and 1% NP-40] and centrifuged. 500 µL of lysate was added to agarose beads and incubated for 2–6 h on a rotator at 4 °C. Samples were incubated overnight at 4 °C with 10 µg of Ago2 antibody (1:10, C34C6, Cell Signalling Technology). The lysate bound antibody solution was added to agarose beads and left on the rotator for 2 h. The supernatant was removed and the pellet was suspended in 200 µL Trizol and an RNA extraction was performed.

### Standard RNA extraction

Hippocampi and cortices were homogenised in 750 µL of Trizol (re-suspended in 200 µL for synaptoneurosomes and AgoIP) and centrifuged at 12,000 *g* for 10 min at 4 °C. Phase separation was performed by adding 200 µL of chloroform to each sample (75 µL for synaptoneurosomes and AgoIP) and vigorously mixing for 15 s before incubating at RT. Samples were centrifuged at 15,600 *g* for 15 min at 4 °C. The upper phase was removed and 450 µL of isopropanol (125 µL for synaptoneurosomes) was added and samples were stored at − 20 °C overnight. Samples were centrifuged at 15,600 *g* for 15 min at 4 °C. 750 µL (200 µL for synaptoneurosomes and AgoIP) of 75% cold ethanol was used to wash the pellet. Samples were centrifuged at 13,300 *g* for 5 min and the ethanol was removed. The pellets were left to dry for 1 h and resuspended in 25 µL (8 µL for synaptoneurosomes and AgoIP) of RNase free H_2_0. Samples were incubated for 10 min at 60 °C with 60 *g* of agitation.

### MiRNA expression

500 ng was reverse transcribed using stem-loop Multiplex primer pools (Applied Biosystems, Dublin, Ireland). We used reverse-transcriptase-specific primers for the mmu-miR-134 (Applied Biosystems miRNA assay ID 00186) and real-time quantitative PCR was carried out on a 7900HT Fast Realtime System (Applied Biosystems) using TaqMan miRNA assays (Applied Biosystems). U6B (Applied Biosystems miRNA assay ID 001093) was used for normalisation. A relative fold change in expression of the target gene transcript was determined using the comparative cycle threshold method (2−∆∆CT).

### Western blotting

Protein was extracted from whole hippocampi and cortices, separated by SDS-PAGE, transferred to nitrocellulose membranes and incubated in the following primary antibodies: Limk1 (1:500, Cell Signalling-3842), Creb1 (1:50, Santa Cruz-271), DCX (1:500, Cell signalling-4604S). Membranes were then incubated in secondary antibodies, and bands were visualised using Supersignal West Pico Chemiluminescence Substrates (Pierce) and captured using a Fuji-Film Las-4000. Thereafter, membranes were stripped (200 mM glycine, 0.1% SDS, 1% Tween 20; pH 2.2), blocked and incubated with GAPDH antibody (1:1000, ThermoFisher Diagnostics-AM4300), secondary antibody and visualised as above. Densitometry was performed using ImageJ software.

### Fluoro-Jade B staining

For analysis of neuronal death, mice were perfused with ice-cold PBS 24 h post SE, and the brains were flash-frozen in 2-methylbutane (at − 80 °C), sectioned at 12 µm in the coronal plane (CM1900 cryostat, Leica) and placed on a glass slide. Frozen tissue was fixed, dehydrated and transferred to a 0.06% potassium permanganate solution for 15 min. Sections were placed into a 0.001% FJB staining solution (in 0.1% acetic acid) for 30 min and rinsed in distilled water followed by histoclear solution. Slides were mounted using DPX mounting medium (Sigma Aldrich, Poole, UK) and examined using a Leica DM4000 microscope. FJB positive cells were counted for individual hippocampal sub-fields (CA1, CA2, CA3 and DG) for each section and an average was taken from three sections per mouse.

### Immunohistochemistry

To determine if high doses of Ant-134 were associated neurotoxicity, brains were assessed using markers for astrogliosis and microgliosis. In addition, we also investigated the effect of Ant-134 on astrogliosis and microgliosis 24 h after KA-induced seizures. Brains were perfused with ice-cold PBS 24 h post ICV injection of Scr/Ant-134 (0.5 nmol; N = 3/group) and flash-frozen in 2-methylbutane. Brains were coronally sectioned at 12 µm (CM1900 cryostat, Leica) and placed on a glass slide. Following quenching and blocking, samples were incubated in primary antibodies GFAP (1:500, Biotechne 29415) and Iba1 (1:500, Wako Chemicals 19,741) overnight at 4 °C. Sections were then incubated in fluorophore-labelled secondary antibodies and DAPI (1:500). Sections were imaged using the Leica microscope (DM4000). For the analysis of potential toxicity with Ant-134 (0.5 nmol), twenty five areas were chosen at random in every image. To determine if Ant-134 protected against astrogliosis and microgliosis, 15 areas were chosen at random in every image. The fluorescent intensities were measured using ImageJ.

### Statistics

Statistical analysis was performed using GraphPad Prism (version 8). Data were tested for normality using Shapiro–Wilk test. Normally distributed datasets were presented as mean ± SEM and analysed with two-tailed Student’s *t*-test or one-way ANOVA with Bonferroni posthoc testing, as appropriate. Non-normally distributed datasets were presented as median ± IQR and analysed using two-tailed Mann–Whitney U test or Kruskal–Wallis test with Dunn’s posthoc testing, as appropriate. The individual tests used for each comparison are specified in the text. α was set to 0.05 for all tests used.

## Supplementary Information

Supplementary Information.

## Data Availability

The datasets generated during and/or analysed during the current study are available from the corresponding author on reasonable request.
