# Supplementary material for: Antagomir-mediated suppression of microRNA-134 reduces kainic acid-induced seizures in immature mice
Source: Sci Rep. 2021 Jan 11;11:340. doi: 10.1038/s41598-020-79350-7 (PMC7801672; doi:10.1038/s41598-020-79350-7)
Supplement: Supplementary file 1 — Supplementary Information. [file 41598_2020_79350_MOESM1_ESM.pdf]

# Supplementary information

***Title**: Antagomir-mediated suppression of microRNA-134 reduces kainic acid-induced seizures in immature mice*

*Authors:* Aoife Campbell, Gareth Morris, Janosch P. Heller, Elena Langa, Elizabeth Brindley, Jesper Worm, Mads Aaboe Jensen, Meghan T. Miller, David C. Henshall and Cristina R. Reschke

## Supplementary Figure 1

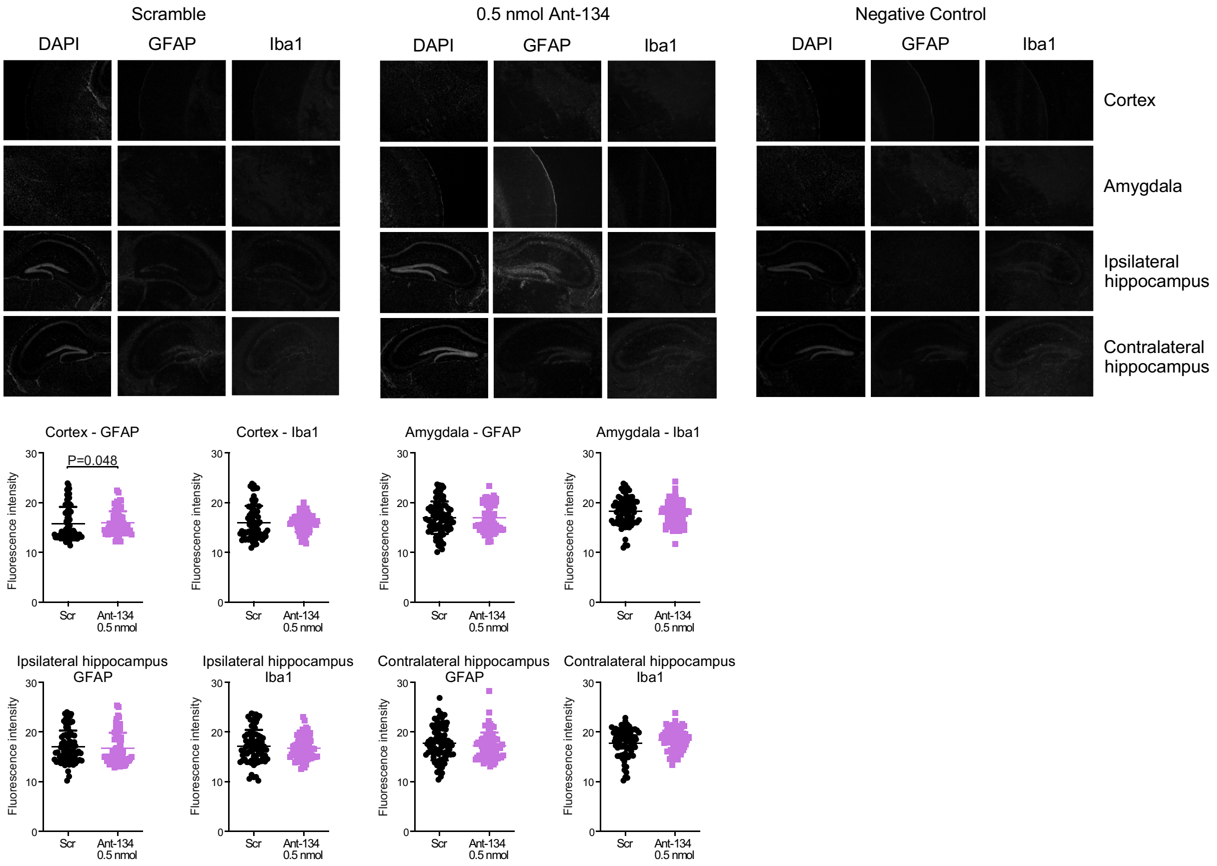


**Supplementary figure 1**: Analysis of microglial activation and reactive astrocytosis suggest minimal toxicity of 0.5 nmol Scr/Ant-134 in naïve P21 mice. Figure shows representative images of tissue sections stained for markers of gliosis from mice pre-treated with Scr/Ant-134 and the negative control. Graphs show the quantification of GFAP and Iba1 in the cortex, amygdala, ipsilateral and contralateral hippocampus. Only a single difference, a slight elevation in GFAP staining, was observed in one region between groups.

**Supplementary figure 2**

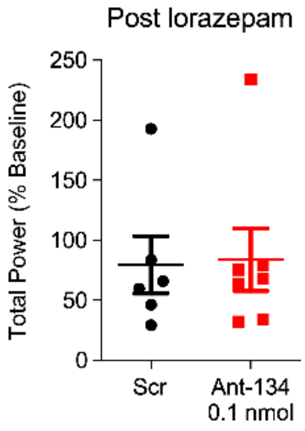


**Supplementary figure 2**: No effect of Ant-134 on post-lorazepam EEG. Graph shows total EEG power following administration of lorazepam in mice pre-treatment with Scr/Ant-134 and subject to KA-induced seizures. Ant-134 does not have an effect on the post-seizure EEG following lorazepam administration. N=6 [scr] or N=7 [Ant-134].

## Supplementary Figure 3

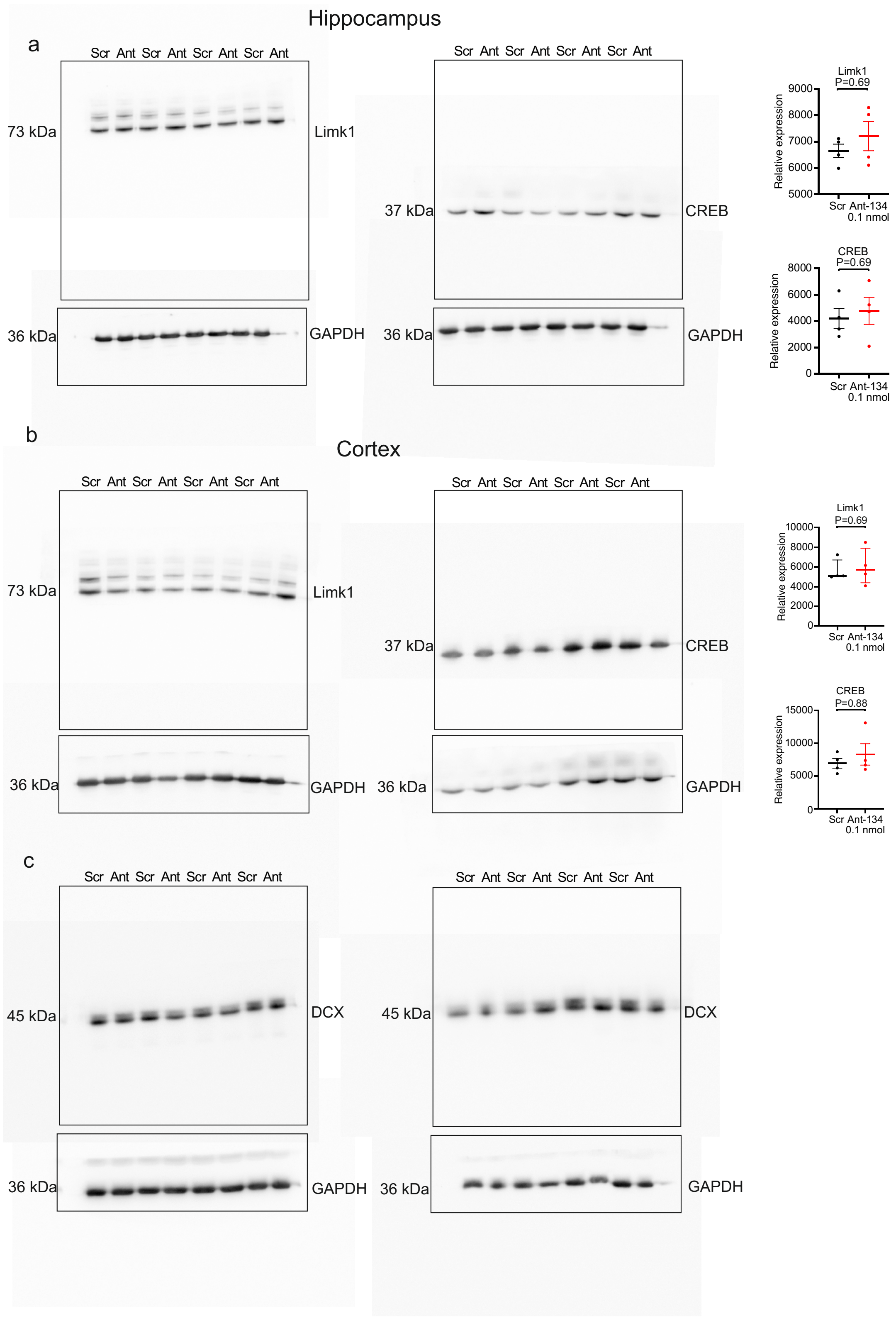

**Supplementary Figure 3:** Western blot analysis of miR-134 targets in mice pre-treated with Scr/Ant-134 and subject to KA-induced seizures. (a) Western blot images and densitometry graphs showing the protein levels of Limk1 and Creb1 in the hippocampus (N=4/group). (b) Western blot images and graphs showing densitometry of protein levels of Limk1 and Creb1 in the cortex (N=4/group). (c) Full-length western blot image showing DCX in the hippocampus and cortex, (N=4/group). Cropped version can be found in Fig 6 g.
